# High Vimentin Expression Associated with Lymph Node Metastasis and Predicated a Poor Prognosis in Oral Squamous Cell Carcinoma

**DOI:** 10.1038/srep38834

**Published:** 2016-12-14

**Authors:** Shuli Liu, Liu Liu, Weimin Ye, Dongxia Ye, Tong Wang, Wenzheng Guo, Yueling Liao, Dongliang Xu, Hongyong Song, Ling Zhang, Hanguang Zhu, Jiong Deng, Zhiyuan Zhang

**Affiliations:** 1 Department of Oral and Maxillofacial–Head and Neck Oncology, Ninth People’s Hospital, Shanghai Jiao Tong University School of Medicine, Shanghai, China; 2 Shanghai Key Laboratory of Stomatology, Ninth People’s Hospital, Shanghai Jiao Tong University School of Medicine, Shanghai, China; 3 Key Laboratory of Cell Differentiation and Apoptosis of Chinese Minister of Education, Shanghai Jiao Tong University School of Medicine, Shanghai, China; 4 Shanghai Key Laboratory for Tumor Microenvironment and Inflammation, Shanghai Jiao Tong University School of Medicine, Shanghai, China; 5 Translation Medicine Center, Shanghai Chest Hospital, Shanghai Jiao Tong University, Shanghai, China

## Abstract

Oral squamous cell carcinoma (OSCC) is a common public health problem worldwide with poor prognosis, which is largely due to lymph node metastasis and recurrence. Identification of specific molecular markers of OSCC with lymph node metastasis would be very important for early and specific diagnosis. In this study, we screened for the potential prognosis markers via unbiased transcriptomic microarray analysis in paired two OSCC cell lines, a lymph node metastatic HN12 cell line and a low metastatic parental HN4 cell line. The results showed that vimentin, with 87-fold increase of expression, was on the top of all upregulated genes in metastatic HN12 cells compared to non-metastatic HN4 cells. Treatment of non-metastatic HN4 cells with TGF-β1 induced epithelial to mesenchymal transition (EMT), with increased vimentin expression as well as enhanced migration activity. Consistently, knockdown of vimentin via siRNA resulted in suppressed invasion and migration activities of HN12 cells, suggesting an essential role of vimentin in EMT-related functions of OSCC cells. Finally, immunohistochemical (IHC) staining analysis showed that high vimentin expression was strongly associated with high lymph node metastases (p < 0.05), and poor overall survival (p < 0.05) in OSCC patients. Thus, high vimentin expression is strongly associated with increased metastatic potential, and may serve as a prediction marker for poor prognosis in OSCC patients.

Oral squamous cell carcinoma (OSCC) has been an important component of the worldwide burden of cancer with about 300,000 new cases each year^1^. Even when the optimal combination of surgical and non-surgical approaches was applied, there were still more than 50% of OSCC patients who experienced relapse, either locally, in regional lymph nodes, or at a distant site^2^. Generally, metastasis to lymph nodes, and the regional lymph nodes were considered as one of the most important adverse prognostic factors for OSCC^3^,^4^. The five-year survival rates for OSCC patients at early stage with localized oral cavity are over 80%, but decreased to 40% when the disease has spread to the neck nodes^5^. Thus, new methods of early detection, risk assessment and early intervention are needed for improvement of the survival of OSCC patients. However, current methods for TNM staging only define primary tumors in two dimensions, and there is still lack of reliable predictors for lymph nodal metastases of OSCC^6^. Therefore, it is necessary to find new molecular markers of metastatic subtype as a supporting method for histological diagnosis of metastatic OSCC.

Epithelial and mesenchymal transition (EMT) has been shown to play a critical role in tumor invasion and metastasis. Many studies show that the invasive ability of malignant tumor cells can be achieved by induction of EMT. Vimentin is a cytoskeletal protein, not expressed in normal epithelial cells, but expressed in mesenchymal cells such as fibroblasts, endothelial cells, and lymphocytes. High vimentin expression has been implicated in OSCC with poor clinicopathological features^7^,^8^,^9^. However, the functional link and the pathological role of vimentin expression in OSCC cells have not been defined. In addition, it is still unclear whether vimentin could serve as a good candidate prognosis marker for metastatic OSCC. In this study, we performed analysis on paired two OSCC cell lines, the parental cell line HN4 with a low metastasis ability, and its metastastic subclone HN12 with a high metastasis rate. HN12 and HN4 cells were derived from the same patient, HN12 was a nodal metastatic subclone from HN4^10^. The genetic backgrounds of the two cell lines are similar except the metastatic potential. We hypothesized that genes differentially expressed in these two OSCC cell lines may be responsible for the difference of their metastatic potential, and may thus serve as a potential marker for predication of lymph node metastasis and patient prognosis. Via a transcriptomic microarray analysis, we found that vimentin was highest upregulated gene in the metastatic HN12 cells in comparison with HN4 cells. Importantly, vimentin is functionally linked to the metastasis-related features of OSCC. Moreover, vimentin expression was significantly correlated with lymph node metastases in OSCC samples. Thus, OSCC patients with vimentin positive staining have high risk for cervical lymph node metastastic potential and should be aggressively treated in clinic.

## Results

### High vimentin expression associated with lymph node metastasis *in vitro*

**Figure 1:**
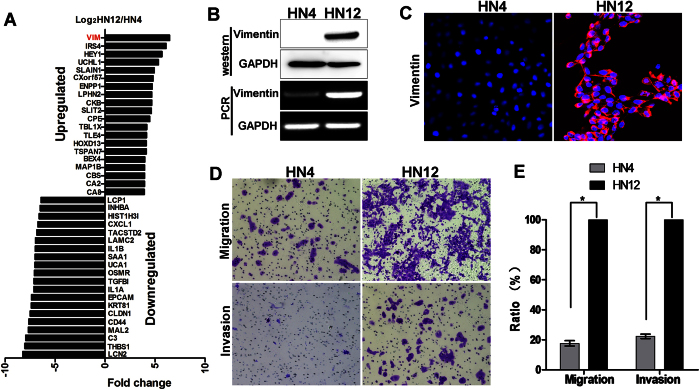
High Vimentin expression is associated with metastatic and invasive activities in oral squamous cell carcinoma cells. (**A**) The microarray analysis between HN4 and HN12 cells. (**B**) Westernblot analysis of vimentin protein levels and RT-PCR analysis of vimentin mRNA levels in HN4 and HN12 cell lines. (**C**) Immunofluorescence staining for vimentin in HN4 and HN12 cells. (**D**) The representative images of the migration and invasion capabilities of HN4 and HN12 cells in the transwell assay. (**E**) Bar graph showed the mean ± SD for the percent of migrated cells from 3 separate experiments, *P < 0.05.

To identify the potential molecular markers related to lymph node metastasis of OSCC, we applied an unbiased transcriptomic microarray method for screening the genes differentially expressed between HN4 and HN12 cells. Using three-fold change as a threshold for the differentially expressed genes obtained from the microarray of two cell lines, we found that total 2322 genes met the criteria, in which 1089 were up-regulated and 1233 were down-regulated in HN12 (data not shown). Among the top 20 up-regulated genes, the vimentin was of the highest, with 87-fold increased expression in HN12 cells compared to HN4 cells (Fig. 1A). The expression level of vimentin in these two cell lines were then validated by Westernblot and RT-PCR, which confirmed the results from microarray analysis (Fig. 1B, Supply Fig. 1). In addition, immunofluorescence (IF) analysis also showed high expression of vimentin in HN12 cells but not in HN4 cells (Fig. 1C).

The process of epithelial to mesenchymal transition (EMT) has been implicated in increased metastasis, migration and invasion of many types of cancer. As vimentin is a marker of mesenchymal cells, its upregulation suggested that EMT is involved in the malignant progression of HN12 cells^11^,^12^,^13^,^14^. To correlate the biological functions with the biochemical differences between the two cell lines, we examined the migratory and invasive activities of HN4 and HN12 cells using a transwell migration assay. The results showed that HN12 cells exhibited significantly higher motility and invasion than did HN4 cells (Fig. 1D and E). Taken together, these results suggest that high vimentin expression in HN12 cells was associated with increased aggressive and metastatic abilities *in vitro*.

### TGF-β induced vimentin as well as EMT in oral cancer

**Figure 2:**
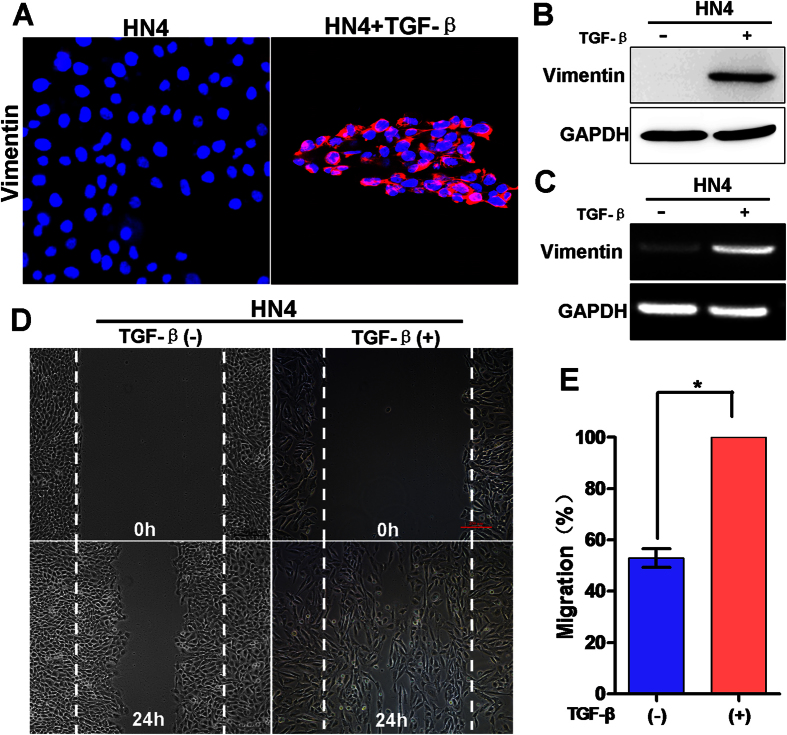
High vimentin expression correlated with TGF-β-induced EMT in oral squamous cell carcinoma cells. (**A**) Immunofluorescence staining for vimentin in HN4 cells treated with or without TGF-β. (**B**) Western blot analysis of vimentin in HN4 cells treated with or without TGF-β. (**C**) RT-PCR analysis of vimentin mRNA levels in HN4 cells treated with or without TGF-β. (**D**) Representative images of Migration of HN4 cells treated with or without TGF-β in assay as described. (**E**) Bar graph showed the mean ± SD for the percent of migrated cells from 3 separate experiments, *P < 0.05.

Transforming growth factor (TGF)-β is a pluripotent cytokine with dual roles in tumorigenesis^15^. Many studies have shown that TGF-β is involved in induction of metastatic and invasive properties in cancer cells, possibly via EMT^16^,^17^. To determine the effect of TGF-β on OSCC cells, we examined vimentin expression in HN4 cells following exposure to TGF-β. TGF-β treatment (5 ng/ml) induced vimentin at both protein level (Fig. 2A and B, Supply Fig. 2) and mRNA level (Fig. 2C). Consistently, the migratory activity, an EMT-associated biological activity, of HN4 cells was also increased following exposure to TGF-β (Fig. 2D and E). This suggested that EMT process was induced in OSCC cells by exposure to TGF-β, which was associated with inducted vimentin and increased invasive ability.

### Knockdown of vimentin inhibits cell migration and invasion in high-metastatic HN12 cells

**Figure 3:**
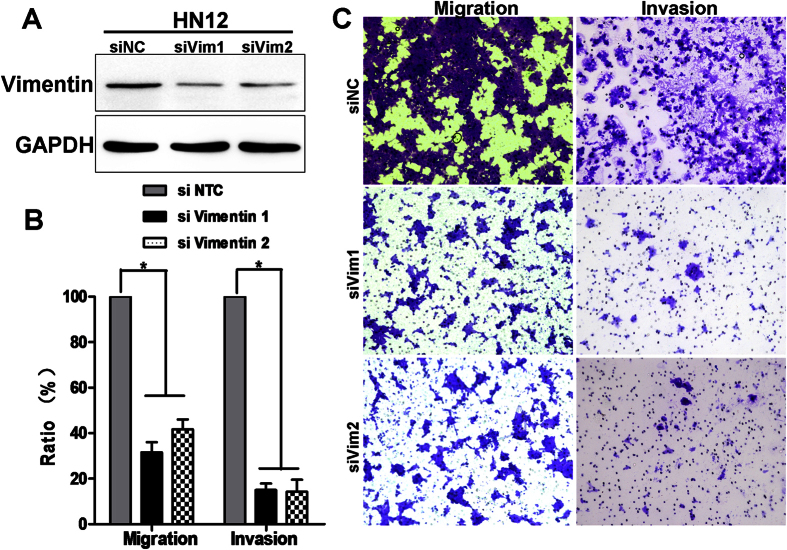
Knockdown of vimentin expression via siRNA resulted in suppressed migration and invasion of HN12 cells. (**A**) Western blot analysis of vimentin in transfected HN12 cells with knockdown of vimentin and control HN12 cells. (**B**) Bar graph showed the mean ± SD percent migrated and invasive cells in HN12 treated with siNC (non-specific control) or siRNA (to vimentin) in 3 separate experiments. (**C**) representative images of migration and invasion of HN12 cells with siNC or siRNA vimentin in transwell assays.

To determine the biological role of vimentin in OSCC cells, we knockdowned vimentin expression in high-metastatic HN12 cells with siRNA and examined the migration and invasion abilities with transwell assy. The knockdown efficiency of endogenous vimentin by siRNA was about 80% (Fig. 3A, Supply Fig. 3). Importantly, knockdown of vimentin resulted in suppressed invasion and migration of HN12 cells (Fig. 3B and C). Taken together, these results suggested that vimentin expression is required for migration and invasion in HN12 cells. Thus, high vimentin expression not only associates with, but also contributes biologically to lymph nodes metastatic features of OSCC cells.

### Lymph node metastasis exhibited high vimentin expression in OSCC patients

**Figure 4:**
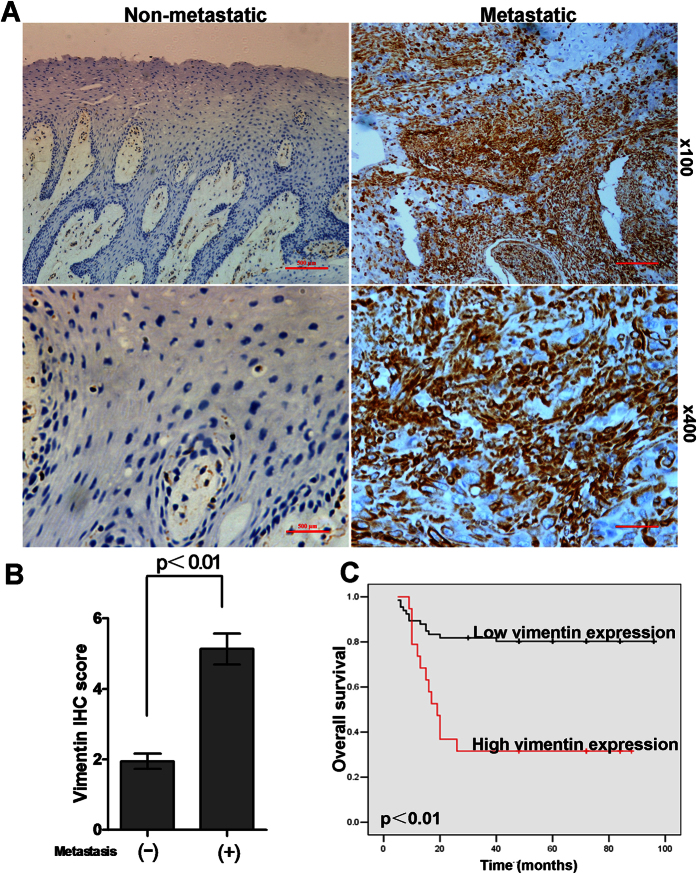
High vimentin expression via immunohistochemical staining is associated with metastasis in oral squamous cell carcinomas. (**A**) tumor samples from OSCC patients without lymph node metastasis showed negative vimentin expression, whereas tumor samples from OSCC patients with lymph node metastasis showed positive vimentin expression. Bar = 500 μm. (**B**) IHC scores of vimentin expression in OSCCs with or without metastasis. Statistically significant differences were detected, using measures analysis of Wilcoxon signed-rank test (P < 0 0.001). (**C**) High vimentin expression significantly correlates with poor survival rate of OSCC patients. The patient’s survival rates of vimentin positive and vimentin negative tumours (P < 0.01) were made using Kaplan–Meier survival test.

**Figure 5:**
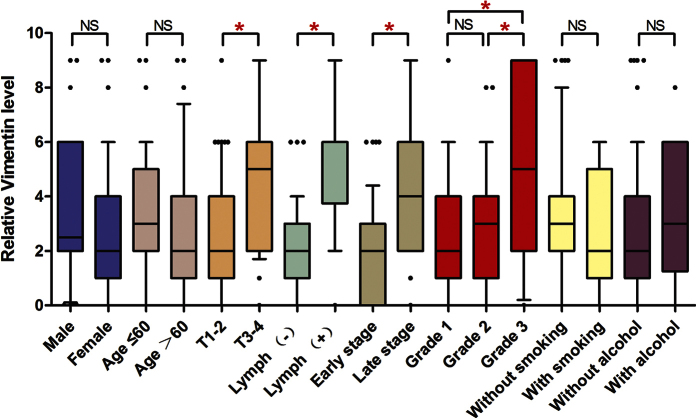
Correlation between Vimentin expression and clinicopathologic features in OSCCs. NS, P > 0.05. *P < 0.05.

To determine if vimentin expression correlates with lymph node metastasis in oral cancer, we examined vimentin protein levels in 85 primary OSCC samples by immunohistochemistry (IHC) staining analysis. The patient demographics and clinicopathological data were shown in Table 1. The results of IHC staining analysis showed that vimentin was expressed in the cytoplasm of mangy tumor cells. Of notion, vimentin protein expression (IHC score) is significantly higher in patients with lymph node metastases (n = 30) than in patients without lymph node metastasis (n = 55) (Figs 4A,B and 5). Taken together, the IHC staining analysis showed that vimentin upregulation was strongly associated with lymph node metastasis in OSCC patients. Thus, high vimentin expression might sever as a risk marker for lymph node metastasis in OSCC.

### High vimentin expression predicated a poor prognosis in OSCC

Regional lymph node metastasis is considered as one of the major risk factors for poor survival of oral cancer patients. To determine the relationship between vimentin expression and survival rate of OSCC patients, we performed survival analysis for the prognostic relation of vimentin expression in OSCC via Kaplan–Meier test. We used vimentin IHC score >4 as standard for high vimentin expression. The result showed that patients with high vimentin expression had a significantly shorter overall survival rate than patients with low vimentin expression (P < 0.01) (Fig. 4C). Taken together, these results demonstrate that vimentin overexpression associated with metastasis to lymph nodes and poor survival rate in OSCC.

## Discussion

In this study, we have identified vimentin as a promising prognosis marker for metastatic OSCC. There are several lines of evidences to support this conclusion. First, vimentin upregulation is on the top of the genes that are differentially expressed in metastatic versus non-metastatic OSCC cells. Second, vimentin, as well as other characters of EMT process, could be induced by TGF-β in OSCC cells. Third, knockdown of vimentin expression resulted in suppressed migration and invasion activities of HN12 cells. And fourth, higher vimentin expression was associated with lymph node metastasis and poor prognosis in clinical OSCC sample analysis. Thus, highly vimentin expression could be served as a risk marker for prediction of cervical lymph node metastasis and poor prognosis.

For OSCC patients, lymph nodes metastasis has been considered as one of the most significant prognostic factors^18^. Despite all available therapeutic strategies, including surgery and combination of radiotherapy & chemotherapy, the five-year survival rate of OSCC is only about 50%^19^. In clinical, the presence of cervical lymph node metastasis was detected by palpation and assisted examination such as computed tomography (CT) and magnetic resonance imaging (MRI). However, the accuracy is greatly limited by resolution and sensitivity of the current method, especially for those pathologically positive lymph nodes in patients staged T1 (d ≤ 2 cm) and T2 (2 cm < d ≤ 4 cm)^20^. To date, optional methods for prediction of patients with stage I/II OSCC (i.e., non-metastatic) that harbor metastatic lymph nodes are limited. And there have been no genetic biomarkers currently applied to clarify this clinical scenario^21^. In this study, we provide the promising marker vimentin as a potential prediction marker for metastatic OSCC and prognosis in OSCC patients. Our results indicated that high vimentin expression was predictive of overall survival rate and lymph node spread in OSCC.

Emerging evidences show that epithelial-mesenchymal transition (EMT) plays an important role in cancer metastasis^22^,^23^. Many transcriptional repressors, such as Snail, Slug, Twist, ZEB1, ZEB2, have been implicated in the regulation of EMT for a variety of cancers, including breast cancer, colon cancer, liver cancer, OSCC and HNSCC^24^,^25^,^26^,^27^. We have previously reported that EMT plays a critical role in lymph node metastasis of OSCC, and the G9a-Snail complex is essential for Snail-induced repression of E-cadherin and EMT in OSCC cells^10^. During the processes of EMT, epithelial cells gain mesenchymal properties and exhibit reduced epithelial features, including decreased intercellular adhesion and increased motility. Vimentin, a mesenchymal-specific protein, is generally not expressed in normal epithelial cells, but induced when cells undergo EMT. Thus, elevated vimentin expression could be considered as a “hallmarker” of EMT. This scenario is true for OSCC. In general, when cells undergo the EMT process, their cytoskeletons are reorganized, accompanied by vimentin overexpression, and an increase in motility. However, how vimentin contributes to EMT-related cancer malignancy is still unknown. Previously, Ching-Yi Liu *et al*.^28^ evaluated the role of vimentin for both the mechanic and tumorigenic approaches in breast cancer cells. They found that vimentin contributed to cytoskeleton organization and focal adhesion stability, which indicated that vimentin maintains cancer cell mechanical homeostasis. With the mechanical modulations generated by vimentin, the EMT-related cancer cells became increasingly organized to resist various stresses generated by the tumor microenvironment, and thus increased in malignancy.

Studies of human epithelial carcinomas, such as breast cancer, hepatocellular carcinoma, colon carcinoma, and prostatic adenocarcinoma, have shown that vimentin expression is correlated with tumor invasion and poor prognosis^29^,^30^,^31^,^32^,^33^. In addition, there were several reports showing that increased expression of vimentin in oral squamous cell carcinoma patient^9^,^34^. However, the pathological roles of vimentin expression in OSCC cells were unclear. In this study, we found that vimentin is on the top of most upregulated gene in metastatic OSCC cells compared to nonmetastatic ones via an unbiased microarray analysis. Importantly, vimentin expression is essential for the increased migration activity of OSCC cells, since knockdown with siRNA resulted in suppressed migration and invasion activities. There is a functional link between vimention expression and the increased metastatic potential. And finally, vimentin expression via IHC staining predicts poor survival rate of OSCC patients. Taken together, the results of this study demonstrate that vimentin is a promising marker for OSCC with lymph node metastasis and prognosis of OSCC.

## Methods

### Primary oral cancer samples

All of the methods were approved by the research medical ethics committee of Shanghai Jiong Tong University and were performed in accordance with the approved guidelines. We obtained archival, formalin-fixed and paraffin-embedded (FFPE) material from surgically resected oral cancer specimens from Ninth People’s Hospital (Shanghai, CHINA), from 2007 to 2011. In total, 85 primary OSCC patients without prior radiotherapy or chemotherapy were enrolled in this study. The age of these patients ranged from 18 to 83 years with an average 57.0 years. Histopathologic diagnosis of each neoplastic tissue was performed according to the World Health Organization criteria by the Department of Oral Pathology, Ninth People’s Hospital of Shanghai. Clinicopathologic staging was determined by the TNM classification of the International Union against Cancer. This study was approved by the Ethics Committee of Shanghai Ninth People’s Hospital, Shanghai Jiao Tong University School of Medicine and carried out according to the recommendations of the Declaration of Helsinki. All the patients involved in this study signed written informed consent in accordance with the institutional guidelines.

### Cell cultures

The OSCC-derived cell lines HN4 and HN12 were cultured in Dulbecco’s modified Eagle’s medium (DMEM; GIBCO, CA) supplemented with 10% FBS, 1% glutamine, and 1% penicillin–streptomycin, and maintained in a humidified atmosphere of 5% CO_2_ at 37 °C.

### Reagents and antibodies

Antibodies against GAPDH were from Santa Cruz Biotechnology, (Santa Cruz, CA). Antibodies for vimentin were from Cell Signaling Technology Inc. (Beverley, MA). HRP-conjugated secondary antibodies were from eBioScience (San Diego, CA). Human recommend TGF-β1 was obtained from R&D system (Minneapolis, MN, USA).

### Microarray analysis

Expression profiling analysis was performed on HN4 and HN12 cells that using Affymetrix U133A microchips. Twenty microgram aliquots of total RNAs were transcribed to first strand complementary DNA (cDNA) using SuperScript II reverse transcriptase (Invitrogen) with an oligo-dT primer that has a T7 RNA polymerase site on the 5′ end, and subsequent second-strand synthesis was carried out to obtain double-strand CDNA. Then, the cDNAs were used in an in vitro transcription reaction in the presence of biotinylated nucleotides to generate single stranded RNAs as recommended by Affymetrix. The biotin-labeled RNAs were fragmented and used for hybridization to Affymetrix human U133 genechips. Data were analyzed using Affymetrix Genechip software. In total, we found that 2322 genes met the criteria, in which 1089 were up-regulated and 1233 were down-regulated in HN12 versus HN4 cell.

### Immunostaining and immunoblotting

Experimental protocols for immunofluorescence staining and immunoblotting follow those previously described^1^. For immunofluorescence staining, cultured cells were rinsed three times with PBS, fixed with 3.7% formaldehyde and permeabilized with 0.1% Triton X-100. After blocking in 1% BSA for 1 hour, cells were incubated with the primary antibody in a moist, 4 °C chamber overnight, washed and then incubated for 1 hour with Alexa Fluor 488 (in the dark) or 594 donkey anti-rabbit IgG (H + L) antibody (Invitrogen, CA, USA) at room temperature. The cells were washed three times with PBS containing 0.02% Tween20 and mounted onto a slide with aqueous mounting medium containing 0.5 mg/ml 40-6-diamidino-2-phenylindole to stain the nuclei. Cells were examined under a fluorescence microscope (Nikon E800) at 400× magnification.

### Reverse Transcriptase Polymerase Chain Reaction (RT-PCR)

The experimental protocol was performed as described previously^34^,^35^. Total RNA samples were extracted with TriPure Isolation Reagent (Roche, Switzerland) and cDNA prepared from 1 mg of total RNA using the SuperScript III System (Invitrogen Life Technologies). The mRNAs levels were determined by RT-PCR, using the following primers: vimentin (F: 5′-GACAATGCGTCTCTGGCACGTCT-3′ and R: 5′-TCCGCCTCCTGCAGGTTCTT-3′); GAPDH (F: 5′-TCCACCACCCTGTTGCTGTA-3′ and R: 5′-ACCACAGTCCATGCCATCAC-3′).

### Immunohistochemistry

Immunohistochemical staining was performed on 4-μm sections of paraffin-embedded specimens with the use of antibody to vimentin. Briefly, 3 μm sections were dewaxed in xylene and hydrated with graded ethanol. Then antigen retrieval was carried out using 0.01 mmol/L citrate buffer (pH 6.0) pressure-cooking, and endogenous peroxidase activity was blocked with 3% hydrogen peroxide for 10 minutes at room temperature. After that the sections were blocked for 1 h at room temperature with normal goat serum. The slides were incubated with the primary antibody (1:200) in a moist chamber for overnight at 4 °C. Upon incubation with the primary antibody, the specimens were washed three times in PBS and visualized using 3,3′-diaminobenzidine detection kit (Dako Cytomation, Denmark). Samples were then counterstained with hematoxylin, a blue nuclear stain. As a negative control, duplicate sections were immunostained with the same concentration of normal rabbit IgG as that of primary antibodies. To quantitate the state of vimentin protein expression, the mean percentage of positive cells was determined in at least five random fields at ×400 magnification in each section. The intensity of the vimentin-immunoreaction was scored as follows: 1+, weak; 2+, moderate; and 3+, intense. At low magnifiation, selected specimens positive cells and uniform distribution area, the mean percentage of random counting 5 unique, non-overlapping high power field of positive cells, according to the percentage of positive cell integral: 0~5% to 0; 6~25% to 1; 26~50% to 2; 51 to 75% to 3 more than 75% to 4. Immunoreactivity score = proportion positive score × intensity score. These judgments were made by two independent pathologists, neither of whom had knowledge or information pertaining to the patients’ clinical status.

### Migration, Invasion and wound healing assay

Experiments were performed as described previously^36^. For the migration assay, cells (5 × 10^5^) were seeded onto the upper chamber in 200 μL of serum-free medium; the lower compartment was filled with 0.6 ml of DMEM media supplemented with 10% of FBS. After 24 h incubation, migrated cells on the lower surface of the filter were fixed and stained using crystal violet; cells on the upper side were removed using a rubber scraper. Fluorescent images were obtained; reported data are counts of migrated cells with experiments performed in triplicate.

### Statistical Analysis

SPSS (Statistic Package for Social Sciences) 13.0 for Windows (SPSS Inc., Chicago, IL, USA) was used to analyze data. The statistical difference of the initial data was analyzed by the non-parametric tests. When the P-value was <0.05, the difference was regarded as statistically significant.

## Additional Information

**How to cite this article:** Liu, S. *et al*. High Vimentin Expression Associated with Lymph Node Metastasis and Predicted a Poor Prognosis in Oral Squamous Cell Carcinoma. *Sci. Rep.* **6**, 38834; doi: 10.1038/srep38834 (2016).

**Publisher's note:** Springer Nature remains neutral with regard to jurisdictional claims in published maps and institutional affiliations.

## Supplementary Material

Supplementary File

## Figures and Tables

**Table 1:** The baseline characteristics of OSCC patients include in the study.

Characteristics	Patients	
	NO.	%
Age, years		
** **≤60	53	62.3
** **>60	32	37.7
Sex		
** **Male	45	52.9
** **Female	40	47.1
T-primary tumor size		
** **T1	24	28.2
** **T2	35	41.2
** **T3	14	16.5
** **T4	12	14.1
N-regional lymph node		
** **Negative	5	64.7
** **Positive	30	35.3
TNM stage		
** **I	25	29.4
** **III	23	27.1
** **III	20	23.5
** **IIV	17	20.0
Histopathological type		
** **Grade 1	36	42.3
** **Grade 2	40	47.1
** **Grade 3	9	10.6
Smoking history		
** **Yes	29	34.1
** **No	56	65.9
Alcohol history		
** **Yes	22	25.9
** **No	63	74.1
Total	85	100
