# Supplementary material for: High Vimentin Expression Associated with Lymph Node Metastasis and Predicated a Poor Prognosis in Oral Squamous Cell Carcinoma
Source: Sci Rep. 2016 Dec 14;6:38834. doi: 10.1038/srep38834 (PMC5155220; doi:10.1038/srep38834)
Supplement: Supplementary File [file srep38834-s1.pdf]

Supplementary Information

High Vimentin Expression Correlated with Lymph Node Metastasis and Predicated a Poor Prognosis in Oral Squamous Cell Carcinoma

Shuli Liu[1,2, #], Liu Liu[1, 2, #], Weimin Ye[1,2, #], Dongxia Ye[1,2], Tong Wang[3,], Wenzheng Guo[3], Yueling Liao[3], Dongliang Xu[3], Hongyong Song[3], Ling Zhang[1,2], Hanguang Zhu[1,2], Jiong Deng[3,4,5] *, Zhiyuan Zhang[1,2] *

[1]Department of Oral and Maxillofacial–Head and Neck Oncology, Ninth People's Hospital, Shanghai Jiao Tong University School of Medicine, Shanghai, China

[2]Shanghai Key Laboratory of Stomatology

[3]Key Laboratory of Cell Differentiation and Apoptosis of Chinese Minister of Education, Shanghai Jiao Tong University School of Medicine, Shanghai, China.

[4]Shanghai Key Laboratory for Tumor Microenvironment and Inflammation, Shanghai Jiao Tong University School of Medicine, Shanghai, China.

[5]Translation Medicine Center, Shanghai Chest Hospital, Shanghai Jiao Tong University, Shanghai, China

***Correspondence to:** Jiong Deng: jiongdeng@shsmu.edu.cn

Zhiyuan Zhang: zhzhy@sjtu.edu.cn

Fig. S1

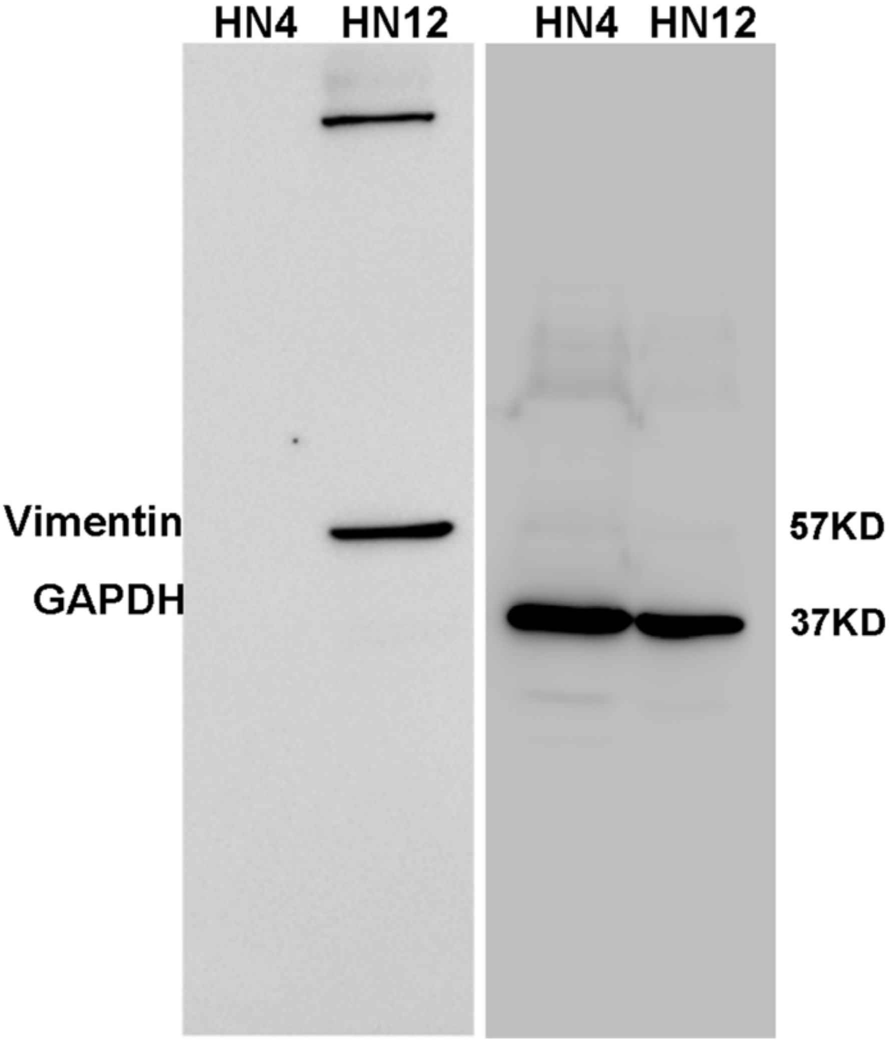

Fig. S1 Westernblot analysis of vimentin protein levels in HN4 and HN12 cell lines.

Fig. S2

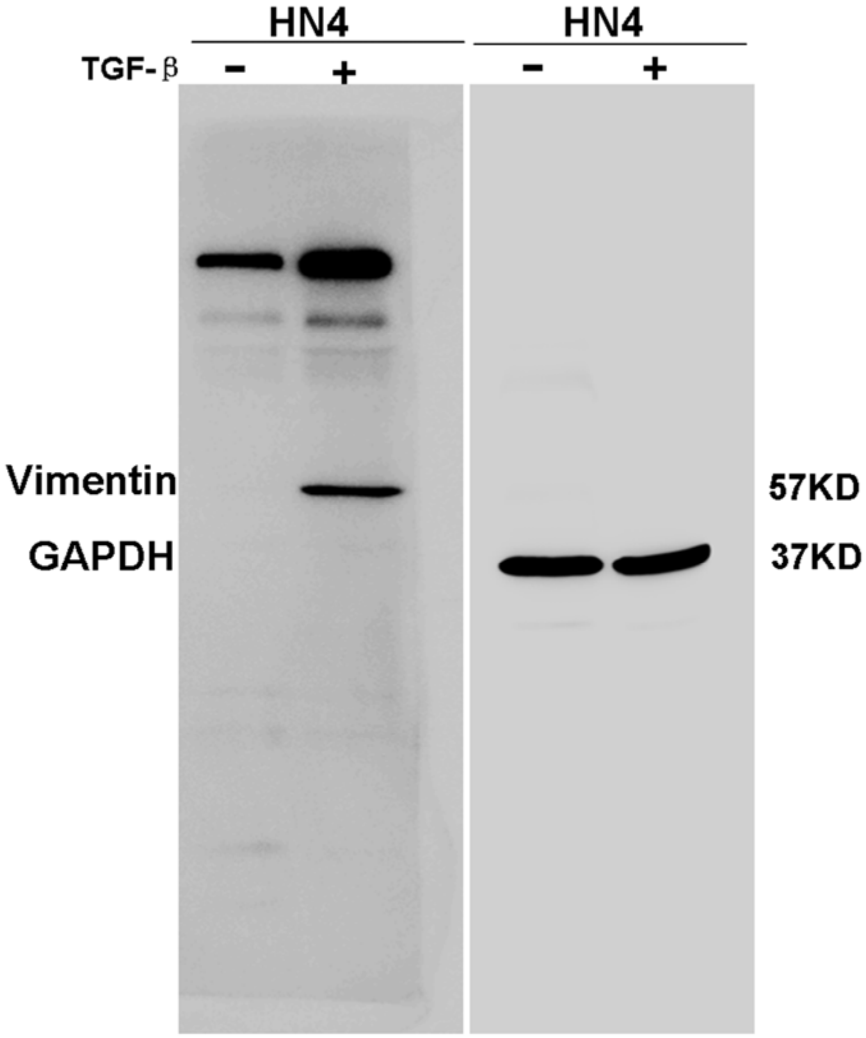

Fig. S2 Western blot analysis of vimentin in HN4 cells treated with or without TGF-β.

Fig. S3

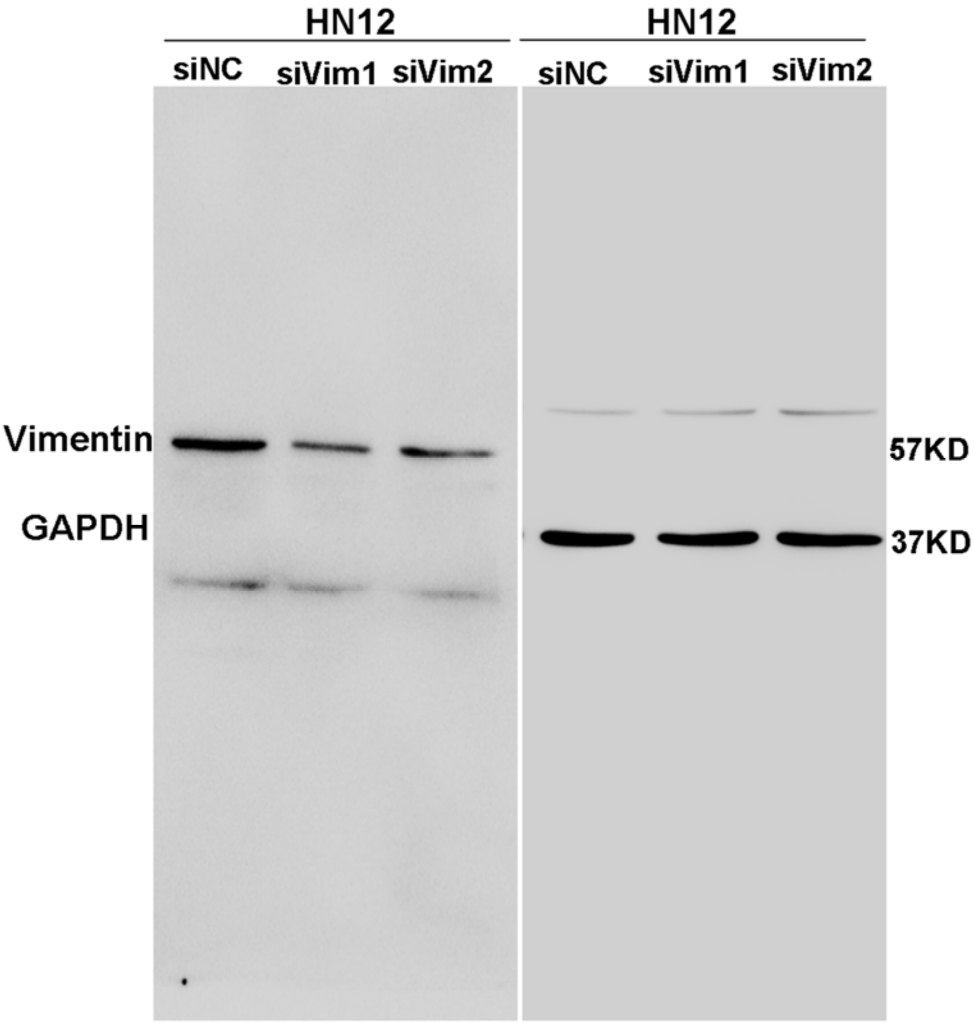

Fig. S3 Western blot analysis of vimentin in transfected HN12 cells with knockdown of vimentin and control HN12 cells.
